# Reply to Çevik et al. Comment on “Inghilleri, G.; Franchini, M. Hypophosphatemia in Patients Receiving Intravenous Iron Supplementation for Iron-Deficiency Anemia: A Narrative Review. *J. Clin. Med.* 2026, *15*, 4748”

**DOI:** 10.3390/jcm15187122

**Published:** 2026-09-14

**Authors:** Giovanni Inghilleri, Massimo Franchini

**Affiliations:** 1 Department of Immunohematology and Transfusion Medicine, Fatebenefratelli Sacco, 20157 Milan, Italy; giovanni.inghilleri@asst-fbf-sacco.it; 2 Department of Hematology and Transfusion Medicine, Carlo Poma Hospital, 46100 Mantua, Italy

We read with great interest the comments by Dr. Çevik and colleagues [1] on our article [2], which we really appreciated as they further enhance the depth of the discussion on IV iron-induced hypophosphatemia.

We fully agree and are aware that hypophosphatemia may occur following intravenous administration of virtually any of the available iron formulations. Indeed, it was first reported in 1982 following the administration of saccharated ferric oxide [3]. In our paper, however, we focused our attention on the two most frequently used third-generation iron formulations, i.e., ferric carboxymaltose (FCM) and ferric derisomaltose (FDI). Among these, FCM has been reported in many studies to be associated with a high frequency and greater severity of hypophosphatemia, both being significantly higher than those observed in patients treated with FDI.

Based on our belief that clinicians should be aware that hypophosphatemia always needs to be considered when patients are treated with IV iron, we reported in Table 1 (of our paper) the data from the PHOSPHARE studies [4,5], showing that the rate of hypophosphatemia following FDI may not be negligible, although it is much lower than that observed following FCM. Regarding the uncertainty of switching to alternative preparations in patients with prior FCM-induced hypophosphatemia, we agree that prospective registries are warranted, although real-world experience and mechanical rationale strongly support switching over re-exposure.

As pointed out by Çevik and colleagues, and in accordance with the available literature, the different iron formulations share a common pathophysiological mechanism leading to hypophosphatemia, namely an increase in intact FGF23, which promotes phosphaturia and inhibits the activation of 25(OH)-vitamin D to 1,25(OH)_2_-vitamin D. However, to the best of our knowledge, the term “6H syndrome” (high FGF23, hypophosphatemia, hyperphosphaturia, hypovitaminosis D, hypocalcemia, and secondary hyperparathyroidism) has been used specifically in relation to FCM-induced hypophosphatemia and, as stated by Blumenstein et al. [6], has not been reported with other iron compounds. This may be attributable to the significantly greater increase in iFGF23 observed following FCM compared with FDI [4,5].

In the second part of their comments, Dr. Çevik and colleagues discuss the recommendations regarding the management of hypophosphatemia reported in our paper, particularly in patients who develop severe hypophosphatemia following IV iron administration. We agree that the recommendations provided may appear somewhat vague; however, this reflects one of the reasons why IV iron-related hypophosphatemia represents a relevant and potentially serious clinical problem: there is currently no standardized treatment for this condition.

This has been clearly stated in a recent expert consensus guideline [7]. Similarly, other recent publications, including those by Rosano et al. [8] and the NATA Consensus [9], do not recommend any specific treatment beyond the use of alternative iron formulations or the avoidance of FCM.

Given the evidence from different studies showing that hypophosphatemia following IV iron, and particularly following FCM, may be very severe (serum phosphate < 1.0 mg/dL), the advice provided by Çevik et al. to adopt, in these cases, treatment protocols used for other FGF23-related phosphate-wasting disorders, such as X-linked hypophosphatemia (XLH), may appear reasonable. In this setting, the administration of vitamin D analogues (alfacalcidol 0–1.5 μg/day, once daily, or calcitriol 0–1.0 μg/day, administered in one or two doses), alone or in combination with phosphate supplements (0–2000 mg/day, distributed throughout the day), was considered conventional treatment before the introduction of burosumab (which is not licensed for IV iron-related hypophosphatemia) [10].

However, this treatment is not without relevant risks, as nephrocalcinosis has been observed in 30% to 70% of treated patients with XLH [10]. Therefore, its use should be limited to more severe or symptomatic cases and should be undertaken by experienced clinicians. Moreover, it must be pointed out that there are currently no clinical studies demonstrating the safety and efficacy of this treatment approach in IV iron-induced hypophosphatemia.

Regarding the comment on the “ambiguity” in the terminology used for vitamin D supplementation, we are once again grateful to Çevik et al. for their extensive and thoughtful discussion of the use of native (25-OH vitamin D) versus active (1,25(OH)_2_ vitamin D), which highlights how the optimal treatment of IV iron-induced hypophosphatemia remains controversial. At present, clinical decisions must largely rely on small studies and pathophysiological considerations. Therefore, specific and evidence-based recommendations cannot yet be provided, which was why we did not discuss in depth the most appropriate form of vitamin D to be used.

Based on pathophysiological considerations and the available published evidence [11,12], in patients with severe hypophosphatemia following FCM administration, particularly with the aim of preventing secondary hyperparathyroidism, we agree with Çevik et al. and support the use of active vitamin D (calcitriol or alfacalcidol), together with close monitoring of serum calcium levels, taking into account the potential risk of nephrocalcinosis and ectopic calcification. Conversely, for the correction of vitamin D deficiency before initiating IV iron treatment, native vitamin D should be used, although no studies have yet demonstrated its clinical efficacy in preventing or mitigating IV iron-induced hypophosphatemia.

We hope that our reply will further contribute to highlighting the clinical relevance of intravenous iron-associated hypophosphatemia and the importance of clinicians being aware of this potentially serious adverse effect. There is an urgent need to define clear and evidence-based guidelines for the management of hypophosphatemia following intravenous iron administration, and we hope that this discussion will stimulate appropriately designed studies aimed at addressing this clinically relevant issue.

## Data Availability

No new data were created or analyzed in this study. Data sharing is not applicable to this article.
